# Evaluation of Genome-Enabled Selection for Bacterial Cold Water Disease Resistance Using Progeny Performance Data in Rainbow Trout: Insights on Genotyping Methods and Genomic Prediction Models

**DOI:** 10.3389/fgene.2016.00096

**Published:** 2016-05-27

**Authors:** Roger L. Vallejo, Timothy D. Leeds, Breno O. Fragomeni, Guangtu Gao, Alvaro G. Hernandez, Ignacy Misztal, Timothy J. Welch, Gregory D. Wiens, Yniv Palti

**Affiliations:** ^1^National Center for Cool and Cold Water Aquaculture, Agricultural Research Service, United States Department of AgricultureKearneysville, WV, USA; ^2^Animal and Dairy Science Department, University of GeorgiaAthens, GA, USA; ^3^High-Throughput Sequencing and Genotyping Unit, Roy J. Carver Biotechnology Center, University of Illinois at Urbana-ChampaignUrbana, IL, USA

**Keywords:** bacterial cold water disease, Bayesian methods, disease resistance, genomic selection, rainbow trout, single-step GBLUP

## Abstract

Bacterial cold water disease (BCWD) causes significant economic losses in salmonid aquaculture, and traditional family-based breeding programs aimed at improving BCWD resistance have been limited to exploiting only between-family variation. We used genomic selection (GS) models to predict genomic breeding values (GEBVs) for BCWD resistance in 10 families from the first generation of the NCCCWA BCWD resistance breeding line, compared the predictive ability (PA) of GEBVs to pedigree-based estimated breeding values (EBVs), and compared the impact of two SNP genotyping methods on the accuracy of GEBV predictions. The BCWD phenotypes survival days (DAYS) and survival status (STATUS) had been recorded in training fish (*n* = 583) subjected to experimental BCWD challenge. Training fish, and their full sibs without phenotypic data that were used as parents of the subsequent generation, were genotyped using two methods: restriction-site associated DNA (RAD) sequencing and the Rainbow Trout Axiom® 57 K SNP array (Chip). Animal-specific GEBVs were estimated using four GS models: BayesB, BayesC, single-step GBLUP (ssGBLUP), and weighted ssGBLUP (wssGBLUP). Family-specific EBVs were estimated using pedigree and phenotype data in the training fish only. The PA of EBVs and GEBVs was assessed by correlating mean progeny phenotype (MPP) with mid-parent EBV (family-specific) or GEBV (animal-specific). The best GEBV predictions were similar to EBV with PA values of 0.49 and 0.46 vs. 0.50 and 0.41 for DAYS and STATUS, respectively. Among the GEBV prediction methods, ssGBLUP consistently had the highest PA. The RAD genotyping platform had GEBVs with similar PA to those of GEBVs from the Chip platform. The PA of ssGBLUP and wssGBLUP methods was higher with the Chip, but for BayesB and BayesC methods it was higher with the RAD platform. The overall GEBV accuracy in this study was low to moderate, likely due to the small training sample used. This study explored the potential of GS for improving resistance to BCWD in rainbow trout using, for the first time, progeny testing data to assess the accuracy of GEBVs, and it provides the basis for further investigation on the implementation of GS in commercial rainbow trout populations.

## Introduction

Bacterial cold water disease (BCWD) causes significant mortality and economic losses in salmonid aquaculture, and methods to control outbreaks are limited (Nematollahi et al., 2003; Barnes and Brown, 2011). We previously reported a family-based, selective breeding program (Silverstein et al., 2009), with the objective of increasing rainbow trout resistance against *Flavobacterium psychrophilum (Fp)*, the etiological agent of BCWD. Resistance to laboratory injection challenge with *Fp* strain CSF259-93 is a moderately heritable trait that responds to selection (Leeds et al., 2010). A selection line, designated ARS-Fp-R, has exhibited higher survival and phenotype stability as compared to reference lines when evaluated on-farm (Wiens et al., 2013). The genetic architecture of resistance is complex (Vallejo et al., 2010) and we previously identified several major resistance QTL in the NCCCWA odd- and even-year rainbow trout selective-breeding populations (Wiens et al., 2013; Vallejo et al., 2014a; Liu et al., 2015b; Palti et al., 2015b). While those loci can be evaluated for marker assisted selection (MAS) following fine-mapping, the complex genetic architecture of BCWD resistance and high genetic variation we discovered in past studies (Vallejo et al., 2014a) led us to hypothesize that a whole genome-enabled selection approach would be a more efficient strategy for improving rainbow trout genetic resistance against BCWD.

Genomic selection (GS) is a relatively recent strategy (Meuwissen et al., 2001) that is revolutionizing plant and animal breeding. This methodology uses high-density marker genotype data that covers the whole genome combined with phenotypic records to compute genomic estimated breeding values (GEBVs) for all genotyped individuals. The GS methodology is chiefly relevant for traits that cannot be directly recorded on the potential breeders or selection candidates including disease susceptibility, carcass and sex-limited traits, and was shown to be highly effective in the dairy cattle industry (Hayes et al., 2009a; VanRaden et al., 2009; Goddard et al., 2011; Wiggans et al., 2011). For aquaculture species like salmonids, the key benefit is that GS enables prediction of individual GEBVs for non-phenotyped fish, and hence exploits within-family genetic variation. In addition to increasing accuracy of selection, GS is expected to decrease inbreeding rate per generation because it enables better differentiation within families and lowers co-selection of sibs (Daetwyler et al., 2007; Dekkers, 2007).

For agricultural livestock species, single nucleotide polymorphism (SNP) arrays or chips have been the platform of choice for whole genome genotyping of at least 50 K SNPs (Matukumalli et al., 2009; Ramos et al., 2009; Groenen et al., 2011); including the recently developed rainbow trout 57 K SNP chip as a new tool available to breeders (Palti et al., 2015a). However, sequencing-by-genotyping methods capable of simultaneous marker discovery and genotyping in many individuals were developed for genetic/genome analyses (Davey et al., 2011). One technique is restriction-site-associated DNA (RAD) sequencing (Miller et al., 2007; Baird et al., 2008) that does not require *a priori* marker discovery or a reference genome sequence. In recent past years, the method of RAD sequencing was widely used in salmonid species for SNP discovery and other genetic/genome analyses (Hecht et al., 2012, 2013; Houston et al., 2012, 2014; Miller et al., 2012; Hale et al., 2013; Narum et al., 2013; Brieuc et al., 2014; Campbell et al., 2014; Gonen et al., 2014; Palti et al., 2014; Liu et al., 2015a).

There is uncertainty about the best computational method for GS. The genomic BLUP (GBLUP) method assumes a polygenic architecture of the trait and uses all the markers data in estimating the genomic relationship G matrix; in contrast, the Bayesian variable selection methods assume that the genetic variance is explained by a reduced number of markers with small-moderate or large effects (Habier et al., 2007; Hayes et al., 2009b; de los Campos et al., 2013; Fernando and Garrick, 2013; Tiezzi and Maltecca, 2015). Based on this assumption, GBLUP is not expected to perform as well as Bayesian variable selection models when the trait is not polygenic and it is controlled by several moderate-to-large effect QTLs. The GBLUP method was modified into the single-step GBLUP method which allows the combination of the pedigree (A) and genomic-derived relationships (G) into a combined relationship matrix (H) (Aguilar et al., 2010; Legarra et al., 2014), and to the weighted single-step GBLUP method which emulates the Bayesian variable selection models by fitting in the multiple regression model selected SNP that explain moderate-large fraction of the genetic variance (Wang et al., 2012).

The accuracy of predicted GEBVs depends on several key parameters including (1) the level of linkage disequilibrium (LD) between the marker loci and the QTL; (2) the number of individuals with phenotype and genotype records in the training population; (3) the degree of relationship between training and testing/validation animals; (4) the average relationship among training individuals; (5) the heritability of the trait, or reliability of breeding values if using de-regressed breeding values; and (6) the distribution of QTL effects (Goddard, 2009; Pszczola et al., 2012). The genetic architecture of the trait coupled with the correct GS model may also have a significant impact on the accuracy of the genomic predictions. Therefore, when evaluating a new trait in a new population and species it is important to compare the accuracy of GEBV predictions from GS models based on single-step GBLUP methods (Aguilar et al., 2010; Wang et al., 2012) as well as Bayesian variable selection models (Fernando and Garrick, 2013; Garrick and Fernando, 2013).

This study was conducted to assess the feasibility of GS for improving BCWD resistance in rainbow trout and compare its accuracy with traditional family-based selective breeding. The objectives of this research were to (1) perform genomic predictions for BCWD resistance using a subset of 10 families from the first generation of the NCCCWA disease resistance breeding line; (2) compare the predictive ability (PA) of classic pedigree-based EBVs with that of GEBV predictions from four different GS models; and (3) compare the PA of GEBVs when using the SNP chip and the RAD genotyping platforms.

## Materials and methods

### Fish rearing and disease challenge

All fish work has been conducted in accordance with national and international guidelines. The protocol for this study was specifically approved by the Institutional Animal Care and Use Committee (IACUC) of the US Department of Agriculture, Agricultural Research Service, the National Center for Cool and Cold Water Aquaculture. All efforts were made to ensure fish welfare and to minimize suffering.

Details of the fish rearing conditions and the 21-day survival study following intraperitoneal injection with the causative agent of BCWD, *F. psychrophilum (Fp)*, was reported elsewhere (Silverstein et al., 2009; Leeds et al., 2010). The procedures of data recording in the disease challenge were also reported elsewhere (Palti et al., 2015b); briefly, the dead fish were removed and recorded daily and fin clipped; the fish health during the study was monitored daily and no unexpected deaths were observed; periodic sampling of the dead fish was conducted to make bacterial cultures and confirm the presence of *Fp* in the dead fish as the likely cause of death; the surviving fish at day 21 post-infection were euthanized in 200 mg L^−1^ of Tricaine methanesulfonate, MS 222 (Sigma) for at least 5 min prior to sampling of their fin clips. The collected fin clips from all fish (mortalities and survivors) were individually kept in 95% ethanol until DNA extraction as previously described (Palti et al., 2006).

### Training and validation fish

The training fish included 10 full-sib (FS) families randomly sampled from a total of 71 pedigreed FS families from year-class (YC) 2005 of the NCCCWA BCWD resistant line (Silverstein et al., 2009; Leeds et al., 2010). The YC 2005 families represented the base generation of the breeding line, and thus had not previously been selected for BCWD resistance. Each family had *n* = 39–80 fish evaluated in the laboratory BCWD challenge in one or two tanks per family with an initial stocking of 40 fish per tank. The total number of training fish with genotypes and phenotypes was 583. The 10 FS families were chosen for use as the training population because non-challenged siblings from these families were used as parents (validation fish) of the subsequent generation, and DNA archives were available for genotyping from the training and validation groups within each family. The validation sample included 53 breeders (sires and dams) that were disease naïve progeny of the 10 training families; each family contributed 2–11 breeders. The breeders or validation fish had family-based EBVs for survival days (DAYS) and survival status (STATUS) estimated using BCWD resistance records measured on their siblings and any collateral relatives among the 71 FS families (*N* = 4492 fish with BCWD resistance phenotypes). In addition, phenotypes from 31 YC 2007 FS progeny testing families (*N* = 1913 progeny with BCWD resistance phenotypes) with both parents from the validation sample were used to calculate the mean progeny phenotype (MPP) for each FS progeny testing family (PTF). This GS study by design ensured a high level of relationship between training and validation fish. A summary of the experimental variables of this GS study for BCWD resistance is presented in Table 1.

**Table 1:** **Experimental variables in pedigree-based and genomic selection analyses for BCWD resistance**.

**Variable**	**PED model^a^**	**Progeny testing^b^**	**ssGBLUP and wssGBLUP^c^**		**BayesB and BayesC^d^**	
			**Training**	**Validation**	**Training**	**Validation**
Number of families	71	31	71	10	10	10
Offspring per family	39–80	38–122	39–80	2–11	39–80	2–11
Genotyped fish	Na^e^	Na	658	53	583	53
Phenotyped fish	4492	1913	4492	Na	583	Na
Pedigree records	4757	Na	4757	Na	Na	Na
Progeny tested breeders^f^	Na	53	Na	53	Na	53

a *Pedigree-based model (PED) fit BCWD records from 2005 families*.

b *The validation fish or potential breeders were mated to generate 31 progeny testing full-sib families*.

c *The single-step GBLUP (ssGBLUP) and weighted ssGBLUP (wssGBLUP) methods used in training models analysis all fish that had genotype and phenotype records and had any type of pedigree relationship (parents, full-sibs, half-sibs, etc.)*.

d *The Bayesian methods BayesB and BayesC used in training model analysis only those fish that had matched genotype and phenotype records without missing data*.

e *Na indicates either non-available or non-needed data cell*.

f *Progeny tested breeders are fish from 2005 families that were used as breeders to generate 2007 families*.

### BCWD resistance phenotypes

The BCWD resistance phenotype DAYS, the number of days to death post-challenge, were recorded for 21 days post-challenge with survivors being assigned a value of 21. Each fish also had a binary survival STATUS record. The BCWD resistance phenotype STATUS had two categories: 1 = the fish died during the 21 days post challenge evaluation period; and 2 = the fish was alive on day 21 post-challenge. In the GS analysis, we used the DAYS and STATUS records from the training fish to first train the GS models and estimate the marker effects, then we estimated the GEBVs for DAYS and STATUS for each validation fish using the estimated marker effects with the training fish.

### SNP genotyping platforms

The training and validation fish with their corresponding parents (YC 2002 and 2003 fish) were genotyped with the recently developed Rainbow Trout Axiom® 57 K SNP array (Chip) as we have previously described (Palti et al., 2015a); the samples were genotyped by a commercial service provider (Geneseek, Inc., Lincoln, NE) following the Axiom genotyping procedures described by the array manufacturer (Affymetrix). For final genotyping calls and quality control analyses we utilized the Affymetrix Power Tools and SNPolisher software applications as we have previously described (Palti et al., 2015a). Each family had between 48,646 and 48,899 genotyped SNPs. The quality control (QC) pipeline filtered out SNPs with significant distortion from the expected Mendelian segregation in each FS family (Bonferroni adjusted to *P* < 0.05) and also removed two training fish that did not have matching genotypes with the parents given in the pedigree (i.e., did not pass pedigree check). After genotype data QC, a total of 49,468 SNPs were included in the raw Chip genotype dataset.

The training and validation fish were also genotyped with ~24 K SNPs generated by sequencing of RAD tag libraries following established procedures in our lab (Palti et al., 2014, 2015b). Genomic DNA from offspring and parents (YC 2005 10 FS families) was digested with restriction enzyme *SbfI*, and RAD sequencing libraries were made as described elsewhere (Palti et al., 2014). Each RAD library that had 30 indexed samples with a unique six-nucleotide barcode for each sample was sequenced (single end 100 bp read) on a single lane of HiSeq 2000; the raw sequences were submitted to the short read archive of GenBank under project accession number PRJNA295850 (Samples: SAMN04090427–SAMN04091127; SRA Accession: SRP063932); and before sequence alignment, we trimmed the six-base barcode at the 5′ end and the last five bases at the 3′ end of each sequence read, and filtered out reads with a cumulative sequencing error probability of more than 20% in the 89 bp read as described elsewhere (Palti et al., 2014). We analyzed the remaining trimmed reads to identify SNPs using Novoalign and Perl scripts as previously described (Liu et al., 2015a). To ensure sufficient sequence reads coverage in the parents, each parent was sequenced twice. The genotype data from parents were called using the bioinformatics pipeline of SNP discovery and genotype calling; and for the offspring genotype calling, the RAD sequences were mapped to the parental alleles of each SNP requiring exact matching in Novoalign as already described (Palti et al., 2015b). The average number of filtered reads per parent was 7.8 M with a range between 5.7 and 12.6 M; for the offspring from the training sample (i.e., fish with phenotypes) the average was 3.0 M with a range between 790 K and 10.0 M per offspring; for the offspring from the validation sample (i.e., fish with EBVs based on siblings and progeny-testing performance) the average was 3.9 M with a range between 2.0 and 9.5 M per offspring. As described elsewhere (Palti et al., 2015b), for each offspring, we required a minimum of four identical sequence reads to call it homozygous for a particular SNP; for heterozygous genotype calls, we required that the total number of reads for the locus (e.g., both alleles) will be ≥4, and the frequency of the minor allele sequence reads (MAF) ≥10%. If both alleles were present in the offspring sample, and the MAF was ≤ 10%, we did not call a genotype for that SNP in that particular offspring and it was recorded as missing data. SNP loci and samples with ≥30% missing data were removed from the final genotype data (SNP/sample calling rate ≥70%). Also, Chi-square goodness-of-fit tests were used to check the genotype segregation ratio (1:1 or 1:2:1) for each SNP, and SNPs with significant Bonferroni-corrected segregation distortion (*P* < 1e-5) were removed from the final genotype dataset. After this genotype data QC, a total of 24,465 RAD SNPs remained in the raw RAD genotype dataset.

Before fitting the GS training models, the total genotyped SNPs were further QC filtered out using QC algorithms implemented in computer program BLUPF90 (Misztal et al., 2015). After this final raw dataset QC, for the Chip SNPs, only those SNPs and samples with genotype calling rate ≥0.90 were included in the GS analysis, with a final effective number of 40,710 SNPs. Likewise, for the RAD SNPs, only those SNPs and samples with calling rate ≥0.70 were included in the GS analysis, with a final effective number of 10,052 SNPs.

### Estimation of EBV with pedigree-based model

For the validation fish, we estimated EBVs for BCWD resistance phenotypes (DAYS and STATUS) using classic pedigree-based model (PED) without genomics or marker genotype data. Family-based EBVs were estimated using BCWD records measured on siblings of the validation fish (YC 2005 families) and any collateral relatives. The phenotypic dataset included DAYS and STATUS records from 4492 fish from 71 FS families (14 paternal half-sib families, 10 maternal half-sib families, and 27 families not nested within a half-sib family), and the pedigree included 4659 records.

Before carrying out PED data analysis, to identify significant predictors of DAYS and STATUS, we executed multivariable regression analysis using mixed linear models that included random family effect, tank, and year fixed effects, and covariate body weight (BW) using STEPWISE model selection with procedure REG from SAS software (SAS, 2007). Then, the experimental variables with significant effect on DAYS and STATUS (potential variables to include in PED model) were assessed for family effect using procedure MIXED from SAS software (SAS, 2007). This latter test is performed to avoid wrongly adjusting response variables for fixed and covariate effects that had significant family effect. At STEPWISE model selection using one-generation BCWD records (YC 2005 families), we found out that BW and tank had significant contribution on the predictive power of DAYS and STATUS. Due to practical restrictions imposed by the disease challenge studies with juvenile fish, the experimental design of our disease challenge experiments confounded tank with family effects; so we decided not to include tank effect in the model of analysis. Next, we found that family had non-significant effect on BW (model tested: BW = mean + family + error) which hints that the covariate BW can be included in the model. Based on these results, we decided that the linear model to estimate EBV should include a population mean effect, random animal effect, continuous covariate BW, and random error effect. The BCWD DAYS and STATUS records were fit into PED linear and threshold models, respectively, using the computer application BLUPF90 (Misztal et al., 2015).

### Estimation of GEBV with Bayesian variable selection models

The Chip or RAD SNP genotype data from the training fish (YC 2005 families) with their corresponding BCWD phenotypic records were used to train the prediction models and estimate marker effects using BayesB and BayesC methods implemented in the software GENSEL (Fernando and Garrick, 2013; Garrick and Fernando, 2013). Before proceeding with the GS analysis, we first performed variance components analysis with BayesC from GENSEL and AIREML implemented in software BLUPF90 (Misztal et al., 2015) to estimate genetic and residual variances for BCWD resistance phenotypes; these estimates of variance components were used as priors in the Bayesian analysis. We performed GS analysis for DAYS using this mixed linear model:

$$\begin{array}{l} y\;=\;1\mu \;+\;Z\alpha \;+\;e \end{array}$$

Where ***y*** is *n* × *1* vector of phenotypic records; **μ** is overall mean; ***Z*** is an *n* × *k* matrix of genotype covariates (coded as −10, 0, or 10) for *k* SNP markers, **α** is a *k x 1* vector of random partial regression coefficients of ***k*** SNPs (additive marker effects), and ***e*** is a vector of residuals.

As outlined in the previous section, we also performed STEPWISE model selection with the training sample used in GS analysis with Bayesian methods which included only fish that had both phenotype an genotype records (*n* = 583) to determine whether family, tank, and weight variables should be included in the model. We observed that tank, BW and family had significant effect on DAYS and STATUS records; nonetheless, we decided not to include the tank effect in the model because tank was confounded with family in the design of our disease challenge studies (Vallejo et al., 2014b). The scatter plot of the first two principal components estimated with software BLUPF90 (Misztal et al., 2015) using the SNP Chip genotype data hinted a population structure. There were nine clusters which represented family groups; we used 10 FS families from which two families shared the same dam parent (maternal half-sib families). We decided not to account for this apparent structure by modeling either family or the two first principal components because it was caused by family genetic effects that are being estimated in the GS analyses. Next, we found that family had significant effect on BW which hints that the BW covariate should not be included in the mixed linear model. The GS analysis of the binary data STATUS was performed using the option for categorical analysis implemented in GENSEL (Fernando and Garrick, 2013; Garrick and Fernando, 2013).

In BayesB and BayesC analyses, the mixture parameter π specifies the proportion of loci with zero effect. So, given a *p* effective number of SNPs, the *k* = (1 − π)*p* markers that are sampled as having non-zero effect are fitted simultaneously in the Bayesian multiple regression model. The mixture parameter π was assumed to be known and defined to meet the condition *k* ≤ *n*; where *n* is the number of training fish. So in the GS analysis with Bayesian methods, we evaluated π values of 0.98, 0.99, and 0.995 with the SNP Chip data; and π values of 0.975, 0.98, 0.99, and 0.995 with the RAD data.

With Bayesian variable selection models BayesB and BayesC, we used a flat prior for the vector β of non-genetic fixed effects, and conditional on the residual variance $\sigma_{e}^{2}$, a normal distribution with null mean and covariance matrix $R\sigma_{e}^{2}$ for the vector of residuals, where R is a diagonal matrix. In addition, $\sigma_{e}^{2}$ was treated as an unknown parameter with a scaled inverse chi-square prior. In BayesB, the prior assumptions are that the marker effects have identical and independent mixture distribution, each marker has a point mass at zero with probability π and a univariate-*t* distribution with probability 1 − π with a null mean, scale parameter $S_{\alpha }^{2}$, and *v*_α_ degrees of freedom (Fernando and Garrick, 2013); and the *t*-distribution in BayesB is equivalent to a univariate normal distribution with unknown null mean and locus-specific variance (Garrick and Fernando, 2013).

In BayesC, the prior assumptions are that the marker effects have identical and independent mixture distributions, where each has a point mass at zero with probability π and a univariate-normal distribution with probability 1 − π having a null mean with variance $\sigma_{\alpha }^{2}$, which has a scaled inverse chi-square prior with $S_{\alpha }^{2}$ scale parameter and *v*_α_ degrees of freedom (Fernando and Garrick, 2013). In addition, in BayesC, a locus-specific variance is assumed which is calculated by using information from the prior and actual data (Garrick and Fernando, 2013).

The computer application GENSEL uses Gibbs sampling methods in all its Bayesian variable selection methods. The BCWD resistance phenotypes were analyzed using 50,000 Markov Chain Monte Carlo (MCMC) iterations from which the first 10,000 samples were discarded as burn-in; from the remaining 40,000 samples, we saved one from every 10 samples (i.e., thinning = 10). To ensure that the MCMC samples were drawn from the full-conditional posterior distributions, we assessed the proper mixing and convergence of the MCMC iterations using the R package CODA (Plummer et al., 2006).

### Estimation of GEBV with single-step genomic BLUP methods

The Chip or RAD SNP genotype data from training and validation fish (offspring from 10 NCCCWA 2005 FS families) with BCWD records measured in training fish and pedigree information on all fish included in this GS study were used to estimate GEBVs for the validation fish (full-sibs of training fish that were not disease challenged) using two methods: (i) single-step genomic BLUP (ssGBLUP; Aguilar et al., 2010; Christensen and Lund, 2010); and (ii) weighted ssGBLUP (wssGBLUP). In wssGBLUP, the weights for each SNP are 1's for the 1st iteration which means that all SNPs have the same weight (i.e., standard ssGBLUP). For the next iterations (2nd, 3rd, etc.), the weights are individual variance of SNP effect estimated in the previous iteration (Wang et al., 2012). In contrast to Bayesian variable selection models, the single-step genomic BLUP methods included in the analysis also progeny from YC 2005 families that had only BCWD resistance phenotype records without marker genotype data: full-sibs of training fish (10 FS families) and 61 additional FS families that were pedigree-related to the 10 FS families that provided training and validation fish (*n* = 4492; Table 1).

Before performing the GS analysis with ssGBLUP and wssGBLUP methods, as a quality check and to have estimates of genetic parameters to use as priors in the Bayesian analysis of the binary STATUS, we performed variance components analysis for DAYS with AIREMLF90 which is implemented in BLUPF90 (Misztal et al., 2015). The variance components analysis and GS analysis for STATUS was performed with the computer application THRGIBBS1F90 which is implemented in BLUPF90 (Misztal et al., 2015). The binary STATUS data were analyzed as categorical data with a threshold model under a Bayesian framework. The MCMC Gibbs sampling scheme included a total of 70,000 iterations; the first 10,000 iterations were discarded as burn-in iterations; then from the remaining 60,000 samples, one from every 20 samples were saved for analysis. This Gibbs sampling scheme collected 3000 independent samples for the analysis. The proper mixing and convergence of these MCMC iterations were also evaluated with the R package CODA (Plummer et al., 2006).

The linear and threshold models to estimate GEBVs for DAYS and STATUS, respectively, included a population mean effect, random animal effect, continuous covariate BW, and random error effect. The mixed-linear model for DAYS and threshold model for the binary STATUS were fitted using a suite of computer applications implemented in the software BLUPF90 (Misztal et al., 2015).

### Predictive ability and BIAS of EBV and GEBV

The predictive ability (PA) of EBV and GEBV, both being estimates of additive genetic effects, was estimated under the assumption that the correlation between mid-parent EBV or GEBV and the MPP for each FS PTF is the best-unbiased estimator of the accuracy of predicted breeding values, given the mixture of 17 FS families nested within 8 paternal half-sib (HS) groups, and 14 FS families not nested within a HS family (total of 31 YC2007 PTFs) in our validation sample (Ødegård et al., 2007; Cheng et al., 2015). Because we did not mate the validation parents to a large, random sample of fish from a common genetic base, but instead mated them to each other, we used the mid-parent BV to account for genetic merit of the mate. So throughout this study, the calculated PA for EBV and GEBV was used as an estimator of prediction accuracy. To estimate mean STATUS and mean DAYS phenotype (MPP) for each PTF, we calculated the mean for each challenge tank, and then calculated the mean of challenge tank means within a family.

In this study, we first estimated EBV and GEBV for each of the *n* = 53 validation sample fish (Data Sheet 1 in Supplementary Material). Then we calculated the mid-parent EBV and GEBV for each of the 31 FS progeny-testing families from YC 2007 (Data Sheet 2 in Supplementary Material). The PA of EBV (*PA*_*EBV*_) was estimated as the Pearson's correlation coefficient of mid-parent EBV with MPP from each PTF, *PA*_*EBV*_ = *CORR*(*EBV, MPP*). To our knowledge, this is the first GS study in rainbow trout that uses empirical progeny testing data to validate the accuracy of genomic predictions.

The bias of EBV prediction was estimated as the regression coefficient of performance MPP on predicted mid-parent EBV (β_*MPP*. *EBV*_). Similarly, the PA of GEBV (*PA*_*GEBV*_) was estimated as the correlation coefficient of mid-parent GEBV with MPP from each PTF, *PA*_*GEBV*_ = *CORR*(*GEBV, MPP*). The bias of GEBV prediction was estimated as the regression coefficient of performance MPP on predicted mid-parent GEBV (β_*MPP*. *GEBV*_). A value of 1.0 for the regression of true breeding value, performance phenotype, or MPP on predicted EBV or GEBV is theoretically expected for unbiased estimates of BV; and a deviation from 1.0 can be interpreted as prediction bias (Saatchi et al., 2013).

## Results

In order to rule out potential errors in the ssGBLUP method and the used statistical model, we performed (1) GBLUP analysis with the current statistical model (phenotype = mean + animal + body weight + error) to ensure that nothing was wrong with the ssGBLUP algorithm; and (2) ssGBLUP analysis with an alternative statistical model (phenotype = tank + animal + body weight + error) to assess the impact of the fixed effect tank in the accuracy of prediction with ssGBLUP. As expected, with the current statistical model, the accuracy of GBLUP (DAYS = 0.41; STATUS = 0.31) was lower than the accuracy of ssGBLUP (DAYS = 0.49; STATUS = 0.46) which suggests that nothing was wrong with the ssGBLUP method. Next, we found that the accuracy of current model ssGBLUP is significantly higher than the accuracy of alternative model ssGBLUP (DAYS = 0.32; STATUS = 0.25) which highlights the adverse effect of including the tank effect in the statistical model. Furthermore, the genetic variance and heritability estimated with the alternative model ssGBLUP $\left(h_{DAYS}^{2}=0.11;h_{STATUS}^{2}=0.33\right)$ had about 100% reduction in comparison to those estimated with the current model ssGBLUP $\left(h_{DAYS}^{2}=0.24;h_{STATUS}^{2}=0.45\right)$. The reduction in genetic variance, heritability and prediction accuracy with alternative model ssGBLUP was due to the confounding of tank with family effects in our disease challenge experimental design; hence, by including the tank effect in the alternative model, the family effect was wrongly accounted twice and depleted the genetic variance. Thus, we confirmed that the statistical model we used in the GS analyses is correct.

### EBV and GEBV predictions for BCWD resistance

For BCWD resistance phenotypes DAYS and STATUS, the pedigree-based EBVs and the GEBV predictions from four GS models using the Chip and RAD genotyping platforms are presented in the Additional File S1.

### Correlation between GEBV estimated with GS models

The correlations between GEBVs for BCWD resistance estimated with four GS models using data from two genotyping platforms are shown in Table 1 in Supplementary Material. The GEBVs were highly correlated (0.81–0.99). As can be expected, the GEBVs estimated with BayesB and BayesC had the highest correlation (0.97–0.99) followed by the GEBVs estimated with ssGBLUP and wssGBLUP (0.91–0.93).

### Heritability of BCWD resistance

The heritability of DAYS and STATUS were 0.31 and 0.48, respectively (Table 2), using the PED model without genomics data.

**Table 2:** **Predictive ability and bias of estimated breeding value (EBV) for BCWD resistance using a pedigree-based model^a^**.

**Phenotype^b^**	**$\sigma_{a}^{2}$^c^**	**$\sigma_{e}^{2}$**	**h^2^**	**Average EBV accuracy^d^**	**Predictive ability^e^**	**Bias^f^**
DAYS	13.73 ± 2.70	30.54 ± 1.58	0.31	0.67	0.50	1.10
STATUS	0.93 ± 0.28	1.00 ± 0.03	0.48	0.67	0.41	0.33

a *The EBVs were estimated using a pedigree-based animal model run with BLUPF90 computer program (Misztal et al., 2015)*.

b *The BLUP analysis included BCWD resistance phenotypes measured on 4492 fish (progeny of 2005 families)*.

c Genetic parameters with their standard error: $\sigma_{\text{a}}^{\text{2}}$ is the additive genetic variance;

*$\sigma_{\text{e}}^{\text{2}}$ is the random error variance; and h^2^ is the trait narrow-sense heritability*.

d *Average EBV accuracy $\left({\bar{\text{R}}}_{\text{EBV}}\right)$ was calculated using EBV accuracy estimated for each validation fish (n = 53)*.

e *The predictive ability (PA) of EBV was calculated using the mean progeny performance (MPP) of 31 progeny testing families that had as parents a pair of validation fish. The PA of EBV was estimated as the correlation of mid-parent EBV with MPP from each progeny testing family, PA_EBV_ = CORR(EBV, MPP)*.

f *The bias of EBV was estimated as the regression coefficient of performance MPP on predicted mid-parent EBV (β_MPP.EBV_)*.

### Predictive ability and bias of EBVs

The PA of EBV for DAYS (*PA*_*EBV*_ = 0.50) was higher than the PA of EBV for STATUS (*PA*_*EBV*_ = 0.41)(Table 2). The bias of EBV for DAYS (β_*MPP*.*EBV*_ = 1.10) was lower than the bias of EBV for STATUS (β_*MPP*.*EBV*_ = 0.33), or DAYS deviated less from 1.0 than STATUS. These results indicate that EBV estimates for DAYS had higher PA and lower bias than those EBV for STATUS.

### Predictive ability and bias of GEBVs

For DAYS, the proportion of genetic variance explained by the markers was $h_{M}^{2}=0.26-0.33$ across GS models and SNP genotyping platforms (Table 3). The PA of GEBVs for DAYS had a range of *PA*_*GEBV*_ = 0.37 − 0.49. The bias of the GEBVs for DAYS had a range of β_*EBV*2.*EBV*_ = 0.32−0.69 which indicates that the GEBVs for DAYS are up-biased.

**Table 3:** **Predictive ability and bias of genomic breeding value (GEBV) for BCWD survival DAYS using four GS models with two genotyping platforms**.

**Genotyping platform^a^**	**GS model^b^**	**Training sample**^**c**^						**Validation sample**^**d**^	
		**Phenotyped**	**Genotyped**	**Effective SNPs^f^**	**π^g^**	**Fitted SNPs^h^**	**$h_{M}^{2}$^i^**	**Predictive ability^j^**	**Bias^k^**
Chip	ssGBLUP	4492	652	40,710	Na^l^	Na	0.29	0.49	0.68
Chip	wssGBLUP^e^	4492	652	40,710	Na	Na	0.29	0.40	0.34
Chip	BayesB	583	583	40,744	0.990	407	0.27	0.39	0.55
Chip	BayesC	583	583	40,744	0.995	204	0.26	0.44	0.63
RAD	ssGBLUP	4492	649	10,052	Na	Na	0.33	0.48	0.63
RAD	wssGBLUP	4492	649	10,052	Na	Na	0.33	0.37	0.32
RAD	BayesB	579	579	10,059	0.975	251	0.28	0.47	0.69
RAD	BayesC	579	579	10,059	0.990	101	0.31	0.46	0.61

a *The effective number of SNPs used was 40,710 and 10,052 from the Chip and RAD genotyping platforms, respectively*.

b *Genomic selection models: single-step GBLUP (ssGBLUP); weighted ssGBLUP (wssGBLUP); Bayesian methods BayesB and BayesC; the GS analysis included only progeny of 2005 families*.

c *The training sample included offspring from 10 full-sib 2005 families each with n = 38–80 offspring; BayesB and BayesC models included only fish that had both genotype and phenotype records (n = 579–583). In contrast, the ssGBLUP and wssGBLUP methods included also non-genotyped fish that had disease records (progeny of 2005 families)*.

d *The validation sample included 53 breeders (offspring of 2005 families that were included in training sample)*.

e *From wssGBLUP, iteration 2 results are presented; iteration 2 yielded higher accuracy GEBVs than iteration 3*.

f *The analysis included SNPs and samples with a calling rate ≥0.70 and 0.90 for the RAD and Chip genotyping platforms, respectively*.

g *Mixture parameter p specifies the proportion of loci with null effect*.

h *Markers that are sampled as having non-zero effect (1−π) are fitted simultaneously in the multiple regression model*.

i *Proportion of genetic variance explained by the markers $\left(h_{M}^{\text{2}}\right)$*.

j *The predictive ability (PA) was calculated using the mean progeny performance (MPP) of 31 progeny testing families that had as parents a pair of validation fish. The PA of GEBV was estimated as the correlation of mid-parent GEBV with MPP from each progeny testing family, PA_GEBV_ = CORR(Midparent GEBV, MPP)*.

k *The bias of GEBV was defined as the regression coefficient of performance MPP on predicted mid-parent GEBV (β_MPP.Midparent GEBV_)*.

l *Na indicates either non-available or non-needed data cell*.

For STATUS, the proportion of genetic variance explained by the markers was $h_{M}^{2}=0.43-0.54$ across GS models and SNP genotyping platforms (Table 4). The PA of GEBVs for STATUS had a range of *PA*_*GEBV*_ = 0.26−0.46. The bias of GEBVs for STATUS had a range of β_*EBV*2.*EBV*_ = 0.13−0.24 which indicates also that the GEBVs for STATUS are up-biased.

**Table 4:** **Predictive ability and bias of genomic breeding value (GEBV) for BCWD survival STATUS using four GS models with two genotyping platforms**.

**Genotyping platform^a^**	**GS model^b^**	**Training sample**^**c**^						**Validation sample**^**d**^	
		**Phenotyped**	**Genotyped**	**Effective SNPs^f^**	**π^g^**	**Fitted SNPs^h^**	**$h_{M}^{2}$^i^**	**Predictive ability^j^**	**Bias^k^**
Chip	ssGBLUP	4492	652	40,710	Na^l^	Na	0.45	0.46	0.24
Chip	wssGBLUP^e^	4492	652	40,710	Na	Na	0.45	0.43	0.14
Chip	BayesB	583	583	40,744	0.995	204	0.44	0.26	0.14
Chip	BayesC	583	583	40,744	0.995	204	0.44	0.31	0.15
RAD	ssGBLUP	4492	649	10,052	Na	Na	0.52	0.42	0.19
RAD	wssGBLUP	4492	649	10,052	Na	Na	0.52	0.40	0.13
RAD	BayesB	579	579	10,059	0.980	201	0.43	0.40	0.23
RAD	BayesC	579	579	10,059	0.980	201	0.54	0.35	0.14

a *The effective number of SNPs used was 40,710 and 10,052 from the Chip and RAD genotyping platforms, respectively*.

b *Genomic selection models: single-step GBLUP (ssGBLUP); weighted ssGBLUP (wssGBLUP); Bayesian methods BayesB and BayesC; the GS analysis included only progeny of 2005 families*.

c *The training sample included offspring from 10 full-sib 2005 families each with n = 38–80 offspring; BayesB and BayesC models included only fish that had both genotype and phenotype records (n = 579–583). In contrast, the ssGBLUP and wssGBLUP methods included also non-genotyped fish that had disease records (progeny of 2005 families)*.

d *The validation sample included 53 breeders (offspring of 2005 families that were included in training sample)*.

e *From wssGBLUP, iteration 2 results are presented; iteration 2 yielded higher accuracy GEBVs than iteration 3*.

f *The analysis included SNPs and samples with a calling rate ≥0.70 and 0.90 for the RAD and Chip genotyping platforms, respectively*.

g *Mixture parameter p specifies the proportion of loci with null effect*.

h *Markers that are sampled as having non-zero effect (1−π) are fitted simultaneously in the multiple regression model*.

i *Proportion of genetic variance explained by the markers $\left(h_{M}^{\text{2}}\right)$*.

j *The predictive ability (PA) was calculated using the mean progeny performance (MPP) of 31 progeny testing families that had as parents a pair of validation fish. The PA of GEBV was estimated as the correlation of mid-parent GEBV with MPP from each progeny testing family, PA_GEBV_ = CORR(GEBV, MPP)*.

k *The bias of GEBV was defined as the regression coefficient of performance MPP on predicted mid-parent GEBV (β_MPP.GEBV_)*.

l *Na indicates either non-available or non-needed data cell*.

Overall, across GS models and genotyping platforms, the PAs of GEBVs for DAYS were higher than those estimated for STATUS, and the bias estimates for DAYS were smaller.

## Discussion

The heritability estimated with the PED model and the proportion of genetic variance explained by the markers estimated with GS model for DAYS were similar to the previously reported heritability for BCWD survival STATUS in this population (Silverstein et al., 2009; Leeds et al., 2010). However, the heritability estimated with the PED model and the proportion of genetic variance explained by the markers estimated with GS model for STATUS were higher than our previous estimates with survival analysis model; here the binary data STATUS was analyzed with a threshold model in the underlying scale of disease liability.

The GEBVs for BCWD resistance estimated with four GS models and across genotyping platforms were highly correlated (0.81–0.99). With the highest correlation between BayesB and BayesC (0.97–0.99) followed by the correlation between ssGBLUP and wssGBLUP (0.91–0.94). These results highlight that the ranking of breeders across the GS models for BCWD resistance in this population is very similar.

On the other hand, the correlation between the pedigree-based model EBV and GEBVs for BCWD resistance was only moderate (~0.60; data not shown) which indicates that EBVs and GEBVs are not similar predictors of animal genetic merit for this trait in this population. Hence, given that moderate correlation between EBVs and GEBVs, the ranking of breeders by the two prediction methods is different and the method with the highest PA and least bias is expected to yield significantly better performance.

The PAs of GEBV for DAYS (*PA*_*GEBV*_ = 0.37−0.49) were higher than those estimated for STATUS (*PA*_*GEBV*_ = 0.26−0.46) reflecting the better fit of the discrete data DAYS to the mixed linear model than the binary data STATUS fit with a threshold model. In this study, the accuracy of genomic predictions for BCWD resistance was in the range of 0.26–0.49 (Tables 3, 4) which is close to those estimated with the PED model (0.41–0.50; Table 2); however, they are still short from the 0.55 maximum realized accuracy that can be expected with a PED model given a heritability of 0.30 (Van Vleck et al., 1987). Given the training sample size used here (*n* = 583) and the heritability of 0.30 for BCWD resistance, we calculated using a deterministic expression (Daetwyler et al., 2008) that genomic predictions with an accuracy of 0.51 are expected if at least 500 independent loci were affecting BCWD resistance; which is close to the best PA of GEBVs achieved in this study. Thus, assuming that there are at least 500 independent loci affecting BCWD resistance and given a heritability of 0.30 for this disease trait, with training samples of 3000 and 10,000 fish we expect to predict GEBVs with accuracy of 0.80 and 0.93, respectively; which are 46 and 69% greater than the expected realized accuracy of PED model EBVs.

### Comparison between EBVs and GEBVs

**Figure 1:**
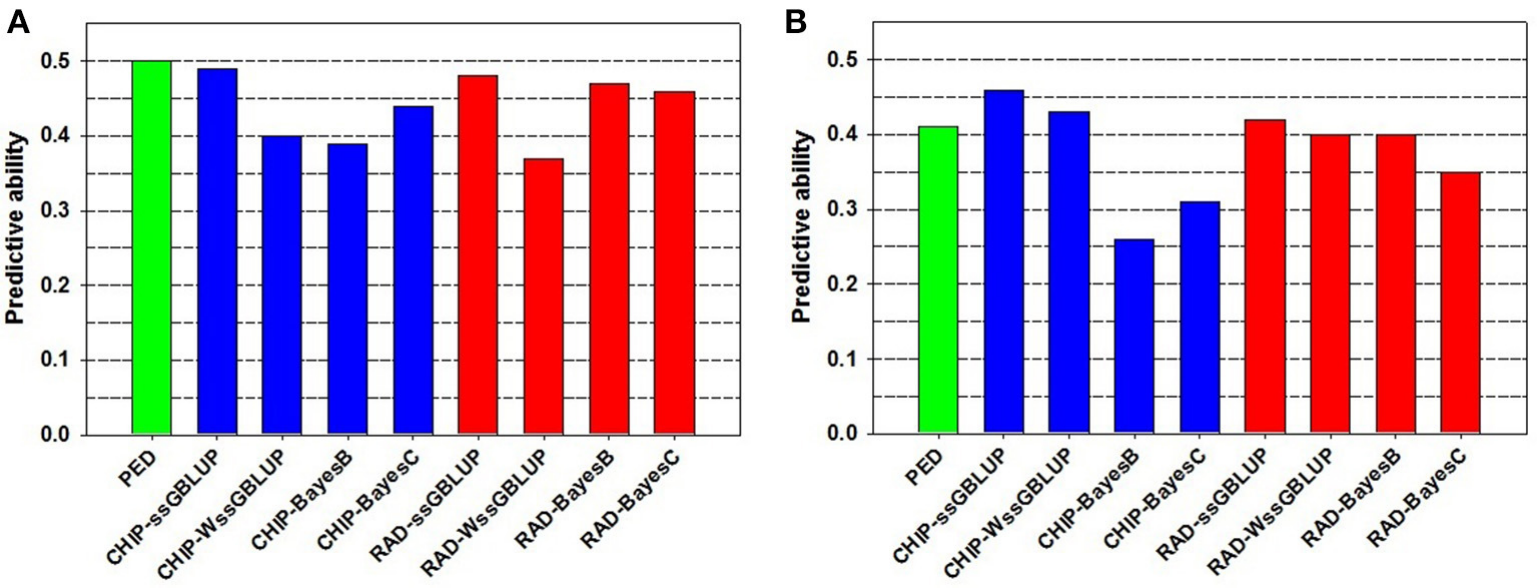
**Predictive ability of estimated breeding value (EBV) and genomic breeding value (GEBV) for BCWD resistance phenotypes: (A) Survival DAYS, and (B) Survival STATUS**.

The PA of EBV for DAYS (*PA*_*EBV*_ = 0.50) (Table 2) is higher than the PA of GEBV for DAYS (*PA*_*GEBV*_ = 0.37−0.49) estimated with four GS models and two genotyping platforms (Table 3, Figure 1A). Conversely, the PA of EBV for STATUS (*PA*_*EBV*_ = 0.41) is lower than the PA of GEBV for STATUS (*PA*_*GEBV*_ = 0.42−0.46) estimated with ssGBLUP at both genotyping platforms (Table 4, Figure 1B).

The bias of EBV for DAYS (β_*MPP*. *EBV*_ = 1.10) (Table 2) is lower than the bias of GEBV for DAYS (β_*MPP*. *GEBV*_ = 0.32−0.69) across GS models and genotyping platforms (Table 3). Likewise, the bias of EBV for STATUS (β_*MPP*. *EBV*_ = 0.33) (Table 2) is lower than the bias of GEBV for STATUS (β_*MPP*. *GEBV*_ = 0.13−0.24) across GS models and genotyping platforms (Table 4).

The PA and bias of the pedigree-based EBVs were similar to those of the best genomic-based GEBVs, which were estimated using the ssGBLUP model (Figure 1, Tables 2–4). Overall, the sample size we used was too small for evaluating the full potential of GS for BCWD resistance in this rainbow trout population. The number of training fish and the number of progeny tested FS families in the validation sample were rather limited in this study. Hence, increasing the sample size of the training and validation populations is expected to increase the PA and accuracy of the GEBV predictions for BCWD resistance in rainbow trout.

### Comparison among GS models

The PA of GEBVs for DAYS estimated with ssGBLUP (*PA*_*GEBV*_ = 0.48−0.49) was higher than those estimated with BayesB (*PA*_*GEBV*_ = 0.39−0.47) and BayesC (*PA*_*GEBV*_ = 0.44−0.46) across genotyping platforms; and the worst accuracy for DAYS was achieved with wssGBLUP using RADs (*PA*_*GEBV*_ = 0.37) which can be attributed to stochastic fluctuations when using relatively small training samples (Table 3). Similarly, the PA of GEBVs for STATUS estimated with ssGBLUP (*PA*_*GEBV*_ = 0.42−0.46) and wssGBLUP (*PA*_*GEBV*_ = 0.40−0.43) were higher than those estimated with BayesB (*PA*_*GEBV*_ = 0.26−0.40) and BayesC (*PA*_*GEBV*_ = 0.31−0.35) across genotyping platforms (Table 4).

Overall, across BCWD phenotypes and genotyping platforms, the GEBVs estimated with ssGBLUP had the highest *PA*_*GEBV*_; and the GEBVs estimated with BayesC had the lowest *PA*_*GEBV*_ (Tables 3, 4, Figure 1). Clearly, the GEBVs estimated with ssGBLUP had higher *PA*_*GEBV*_ than those estimated with wssGBLUP. The method BayesB outperformed BayesC marginally by about 0.06 *PA*_*GEBV*_ units (Figure 1).

Across BCWD phenotypes and genotyping platforms, the GEBVs calculated with ssGLBUP were the least biased or had smallest departure from 1.0 (Tables 3, 4). In contrast, the GEBVs estimated with wssGBLUP were the most biased or had largest departure from 1.0. The GEBVs estimated with BayesB and BayesC had intermediate bias to that of ssGBLUP and wssGBLUP. Between the Bayesian methods, BayesB provided less biased GEBVs than BayesC.

The most accurate ssGBLUP GEBV (DAYS with Chip) had a PA of 0.49, which was only slightly better than the best BayesB and BayesC estimators with PA = 0.47 and 0.46, respectively. Interestingly, the accuracy of the GBLUP models was higher with the Chip genotyping platform, while the Bayesian models' accuracy was better with the RAD platform. The ssGBLUP outperformed wssGBLUP across all phenotypes and genotyping platforms. The wssGBLUP accuracy was slightly better than the Bayesian models for the STATUS phenotype, but less accurate with the DAYS phenotype. BayesB accuracy was better than BayesC with the RAD genotyping platform, but BayesC was more accurate with the Chip platform.

Previously we have shown that the genetic architecture of BCWD resistance in this rainbow trout population is controlled by oligogenic inheritance of few moderate-large effect QTL and many genes/loci each with a small effect (Vallejo et al., 2010, 2014a; Liu et al., 2015b; Palti et al., 2015b). Thus, given that genetic architecture, it seems that GS models that use pedigree and phenotype records with marker genotype data in a single-step GS analysis (Aguilar et al., 2010; Legarra et al., 2014) can yield GEBVs with higher accuracy than methods based on shrinkage or variable selection models (Garrick and Fernando, 2013) that fit in the Bayesian multiple regression model markers with mostly moderate-large effect. However, we caution that the advantage of ssGBLUP over Bayesian variable selection models in this study was very small and hence should be validated using larger training and validation samples.

### Comparison between Chip and RAD platforms

The Chip genotyping platform had GEBVs with higher *PA*_*GEBV*_ than those from RAD genotyping platform when using ssGBLUP and wssGBLUP (Tables 3, 4, Figure 1). However, when using Bayesian methods, the RAD had GEBVs with higher PA than the Chip genotyping platform.

Overall, across BCWD phenotypes and GS models, the Chip platform yielded GEBVs with lower bias than those estimated with RAD (Tables 3, 4). For both BCWD phenotypes, the RAD platform had GEBVs with lower bias than those estimated with Chip only when using BayesB.

The numbers of effective SNPs after genotype data QC were about 40 and 10 K for the Chip and RAD genotyping platform, respectively. Hence, it was somewhat surprising that the RAD platform (*PA*_*GEBV*_ = 0.35−0.48) was as efficient as the Chip platform (*PA*_*GEBV*_ = 0.26−0.49) in accuracy of genomic predictions. Another study also reports that the accuracy of GEBVs using RAD and SNP marker genotype data were similar when large numbers of markers were genotyped and the read's depth per individual was ≥1x (Gorjanc et al., 2015). RAD or similar genotype-by-sequencing methods offer an attractive option for species with less developed genome resources lacking the availability of an affordable high-density SNP chip. However, the SNP Chip is higher throughput than the RAD platform, and the RAD sequencing SNPs are more family and population specific which does not facilitate generating high density panels with common SNPs that are informative across-families and populations. In addition, the bioinformatics pipeline for the SNP Chip is more robust and much easier to implement, and hence we find that the chip platform is more practical for large scale genome genotyping studies.

We hypothesize that the relatively low marker density RADs were as efficient as the SNP Chip due to the high extent of long-range LD in our rainbow trout disease resistance line. The high extent of long-range LD was likely generated by the high level of admixture in this population which had as founders four distinct domesticated strains (Johnson et al., 2007; Silverstein et al., 2009); and this admixture also simultaneously reduces the short-range LD in the population. These population genetic events likely decreased the relative benefit of high-density SNP data, as a fairly larger fraction of the existing LD can be captured even by sparse marker panels, possibly explaining the good performance of the RADs at lower marker density than the SNP Chip. A similar phenomenon of high extent of admixture induced long-range LD that enabled efficient GS at relatively low marker density was reported in farmed salmonid populations (Ødegård et al., 2014). Another factor possibly contributing to the relative success of the RAD platform in this study, is that many of the RAD SNPs are family-specific and hence some genome regions that harbor QTL may be better represented in some of the families by the RAD genotype data. The question then is whether the RAD SNP data set for this population detects the same QTL and at the same signal intensity as the Chip SNPs, and to answer that question we are currently conducting genome-wide association analysis with the two datasets.

### Additional remarks and comparison with other GS studies

A shortcoming of this study is the *post-hoc* assessment using limited number of archived training and validation samples and using a less-than-ideal design at the training and validation steps. However, we should highlight that this is a proof-of-principle study that aims to give insight about the relative performance of several models and genotyping platforms when applied to two disease resistance phenotypes with different statistical properties, rather than being the definitive evaluation of GS compared to PED-based EBVs. Furthermore, this study provides new and more comprehensive empirical data that furthers (or at least validates) our understanding about the genetic architecture of BCWD resistance in rainbow trout.

In comparison to dairy cattle and other farmed animals, one of the main challenges for implementing GS in family-based breeding programs with salmonid species is the high number of potential selection candidates and the low value of the selection candidates in comparison to the genotyping cost. Nevertheless, the sib-testing scheme in salmonid disease resistance breeding programs can be redesigned to capitalize on the ability of GS to increase accuracy of genomic prediction and rate of genetic gain. Alternative strategies could involve the pre-selection of candidates for genotyping as suggested elsewhere (Sonesson and Meuwissen, 2009; Lillehammer et al., 2013; Ødegård and Meuwissen, 2014).

To this end, for rainbow trout GS, we suggest combining a first step of traditional sib-testing disease challenge evaluations to pre-select for disease resistant families, followed with a second step of selective genotyping individuals from the pre-selected families. In this GS scheme, the disease phenotype and marker genotype records from pre-selected families can be used as the training sample to train the GS prediction models, to then predict GEBV for each genotyped selection candidate or disease naive sibs from pre-selected families at the first step.

The unique features of genome-enabled selection such as to increase accuracy of animal EBV prediction and response to selection while not increasing the rates of inbreeding are one of the key benefits of GS in livestock species. The ability of GS to reduce rates of inbreeding has been reported in poultry (Wolc et al., 2015), and a much larger reduction in rate of inbreeding was reported in aquaculture breeding programs due to sib-testing for both sexes (Sonesson and Meuwissen, 2009). The core reason for the decreased rates of inbreeding with GS is that genomic data offers information on Mendelian sampling terms which diminishes the emphasis placed on family selection and thus reduces the correlation of EBVs among family members and likelihood of co-selecting relatives (Daetwyler et al., 2007). Moreover, selection is usually performed over several generations, and the Bulmer effect would diminish between-family variation in the population. Consequently, the capability to use within-family genetic variation will be more important for forthcoming generations. Hence, because traditional sib-selection breeding schemes with salmonids do not exploit within-family genetic variation, the relative benefit of GS is projected to rise if selection is applied over many generations (Ødegård and Meuwissen, 2014).

A major challenge for applying GS in commercial aquaculture breeding programs is collecting large training or reference population which is necessary for accurate estimation of marker effects (Goddard and Hayes, 2009). In this study, the number of training fish was limited so increasing the size of the training population would be expected to increase further the accuracy of GEBV for BCWD resistance in this rainbow trout disease resistance line. Finally, this study provides the basis for additional research on the use of GS in rainbow trout populations, including the potential for its implementation in the rainbow trout industry.

## Conclusion

The results of this study show the potential utility of GS for exploiting the within-family genetic variation in rainbow trout family-based selective breeding programs. Here, the number of training fish was limited so increasing the size of the training population would be expected to increase the accuracy of genomic prediction for BCWD resistance in this rainbow trout population. We expect that by using a larger training sample size with improved GS experimental design, we can exploit the advantage of GEBVs for BCWD resistance in rainbow trout over the classical sib-selection breeding scheme that does not exploit within-family genetic variation. Thus, this study provides the basis for further investigation on the implementation of GS in commercial rainbow trout populations.

## Author contributions

YP, TL, and RV conceived and planned the study; YP, GW, and TW coordinated and performed the disease challenges and samples collection; YP coordinated and performed samples processing, RAD libraries preparation, and genotyping; AH performed RAD libraries sequencing, GG performed genotype data quality control and bioinformatics filtering and developed a database pipeline to assemble genotype and phenotype records; TL provided pedigree records, conducted quantitative analyses to estimate EBVs, and identified the fish for genotyping based on pedigree and phenotype records; RV executed the statistical data and genomics analysis and wrote the first draft of this document; BF and IM provided support in single-step GBLUP analysis. All authors read and approved the final manuscript.

### Conflict of interest statement

The authors declare that the research was conducted in the absence of any commercial or financial relationships that could be construed as a potential conflict of interest.
