# Supplementary material for: Evaluation of Genome-Enabled Selection for Bacterial Cold Water Disease Resistance Using Progeny Performance Data in Rainbow Trout: Insights on Genotyping Methods and Genomic Prediction Models
Source: Front Genet. 2016 May 27;7:96. doi: 10.3389/fgene.2016.00096 (PMC4883007; doi:10.3389/fgene.2016.00096)
Supplement: Supplementary file 1 [file Table1.DOCX]

**Table S1** Correlation^1^ between genomic breeding values for BCWD resistance estimated with four genomic selection models^2^ using data from two SNP genotyping platforms.

| **Correlation of GEBVs** | **Chip^3^** | |  | **RAD^3^** | |  |
| --- | --- | --- | --- | --- | --- | --- |
|  | **DAYS^4^** | **STATUS^4^** |  | **DAYS^4^** | **STATUS^4^** | |
| CORR(GEBV_BayesB_, GEBV_BayesC_) | 0.99 | 0.97 |  | 0.99 | 0.98 | |
| CORR(GEBV_BayesB_, GEBV_ssGBLUP_) | 0.88 | 0.87 |  | 0.89 | 0.88 | |
| CORR(GEBV_BayesB_, GEBV_wssGBLUP_) | 0.81 | 0.85 |  | 0.85 | 0.86 | |
| CORR(GEBV_BayesC_, GEBV_ssGBLUP_) | 0.90 | 0.93 |  | 0.87 | 0.91 | |
| CORR(GEBV_BayesC_, GEBV_wssGBLUP_) | 0.84 | 0.90 |  | 0.88 | 0.90 | |
| CORR(GEBV_ssGBLUP_, GEBV_wssGBLUP_) | 0.92 | 0.93 |  | 0.91 | 0.91 | |
| **Mean** | **0.89** | **0.91** |  | **0.90** | **0.91** | |

^1^Pearson correlation between genomic breeding value (GEBV) estimated with different GS models in validation animals (*n* = 53).

^2^Genomic selection (GS) models: BayesB, BayesC, single-step GBLUP (ssGBLUP) and weighted ssGBLUP (wssGBLUP).

^3^The effective number of SNPs used was 40,710 and 10,052 from the Chip and RAD genotyping platforms, respectively.

^4^BCWD resistance phenotypes: Survival days (DAYS) and binary survival status (STATUS).
